# Food Safety in Hydroponic Food Crop Production: A Review of Intervention Studies to Control Human Pathogens

**DOI:** 10.3390/foods14132308

**Published:** 2025-06-29

**Authors:** Melanie L. Lewis Ivey, Abigail Aba Mensah, Florian Diekmann, Sanja Ilic

**Affiliations:** 1 Department of Plant Pathology, College of Food, Agricultural, and Environmental Sciences-Wooster, The Ohio State University, Wooster, OH 44691, USA; 2 Human Nutrition, Department of Human Sciences, College of Education and Human Ecology-Columbus, The Ohio State University, Columbus, OH 43210, USA; mensah.100@osu.edu; 3 University Libraries-Columbus, The Ohio State University, Columbus, OH 43210, USA; diekmann.4@osu.edu

**Keywords:** food safety interventions, DWC (deep water culture), NFT (nutrient film technique), fresh produce, foodborne pathogens, hydroponics, CEA (controlled environment agriculture)

## Abstract

The production of hydroponic fresh produce presents unique food safety and intervention challenges. A systematic approach was used to map and characterize the evidence on hydroponic food safety. Quantitative data describing the effectiveness of intervention studies were extracted, synthesized, and assessed for quality. A search of electronic databases yielded 131 relevant papers related to hydroponic food safety. Thirty-two studies focusing on food safety interventions reported 53 different interventions using chemical (n = 39), physical (n = 10), multiple-hurdle (n = 2), and biological (n = 2) approaches. Human pathogen indicators and surrogates were most often studied (n = 19), while pathogenic strains like *Salmonella* spp. (n = 9), Shiga toxin-producing *Escherichia coli* (STEC) (n = 5), *Listeria monocytogenes* (n = 2), and viruses (Hepatitis A virus (HAV), n = 1; norovirus (NoV), n = 1) were studied less frequently. Of fourteen articles (43.8%) investigating pre-harvest interventions, most (42.9%) did not specify the hydroponic system type. Gaps remain in the available evidence regarding the efficacy of interventions for controlling human pathogens in near-commercial hydroponic systems. The quality assessment revealed a significant lack of detailed reporting on methods and outcomes, making it difficult to translate the findings into practical recommendations for the industry; therefore, this review provides recommendations for the scientific community to improve future research design and reporting in this field.

## 1. Introduction

**Figure 1:**
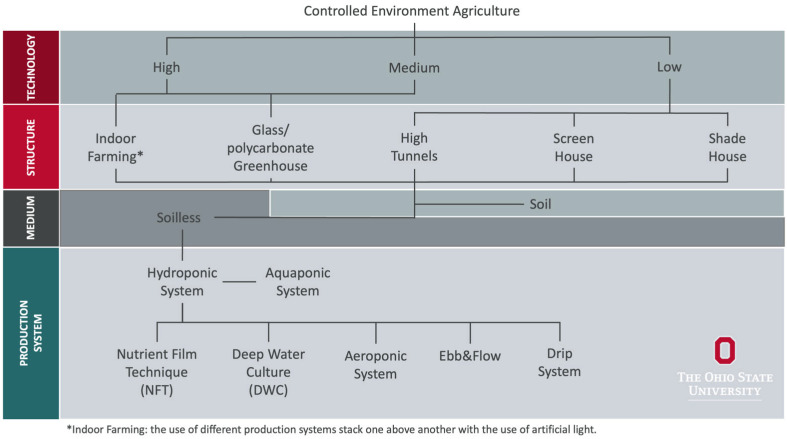
Classification of controlled environment agriculture (CEA) based on a technology level, infrastructure, growing medium, and production system (https://ohioline.osu.edu/factsheet/hyg-5819, accessed on 8 January 2025).

Controlled environment agriculture (CEA) includes both soil and soilless crop production systems regulated under different levels of technology for crop growth (Figure 1). Hydroponic, defined under the umbrella of CEA, is a soilless cultivation system most commonly used for leafy green production, where crops are grown in nutrient-rich water solution using advanced technologies to enhance crop growth within controlled indoor and greenhouse structures. Hydroponic fruit and vegetable production is rapidly expanding due to demand for year-round locally sourced fresh produce. Currently, the market is valued at approximately USD 961.8 million in the United States (US) and is projected to grow at a rate of 10.7% annually [1]. However, concerns about food safety and the contamination of hydroponic produce with human pathogens are growing. Recently, several foodborne illnesses and recalls linked to hydroponic leafy greens have been reported [2,3,4]. In a 2021 multistate outbreak of hydroponic leafy greens, 31 consumers contracted salmonellosis, and four were hospitalized due to salmonellosis [5]. The contamination was traced back to water sources and other food safety practices in the chain of custody [6]. Multiple recalls across the US have been issued since due to possible contamination of hydroponic produce with human pathogens like *Salmonella* spp. and *Listeria monocytogenes* [7,8].

Hydroponic farming presents unique food safety challenges [9,10,11]. In hydroponic systems, crops are constantly exposed to the nutrient solution, providing a pathogen transmission route. If pathogens enter the nutrient solution system, either through contaminated substrate, source water (surface, underground, or municipal), workers, or surface materials, they can cross-contaminate the edible parts of the crops [11]. In contrast to field production, plant exudates—organic compounds secreted by roots—do not remain in the rhizosphere but leach into and circulate in the nutrient solution, providing a favorable environment for bacterial growth and biofilm formation [12].

Additionally, the nutrient solution is aerated, making it oxygen-rich, which can significantly influence microbial dynamics, leading to potential microbial risks [13]. Due to the lack of clean breaks in production, pathogen biofilms can be established on hydroponic surfaces [10]. In the absence of adequate mitigation strategies, these biofilms pose a severe threat to the food safety of hydroponic crops.

Food safety guidelines for fresh produce (Food Safety Modernization Act (FSMA) Produce Safety Rule) [14] were developed for soil-based systems and do not adequately address the specific needs of hydroponic production. Very few strategies, actions, or treatments (food safety interventions) to reduce or eliminate food safety hazards are available for hydroponic systems. The limited nature of the evidence on food safety strategies in hydroponics, dispersed across various disciplines, makes it challenging to form comprehensive recommendations for industry.

Food safety interventions to mitigate microbial risks, encompassing chemical, biological, and physical approaches, have been studied in research laboratories and the post-harvest value chain [15,16,17]. However, evidence of their effectiveness remains unclear, inconsistent, or insufficiently representative of the risks in hydroponic production. This further complicates the development of evidence-based recommendations for the hydroponic industry. Therefore, the synthesis of scientific evidence to effectively prevent and mitigate human pathogens in hydroponic production is needed.

A scoping review approach was used to synthesize, assess the extent and content, and map the existing body of literature on food safety in hydroponic fresh produce. Further, we identified and characterized the studies that reported the effectiveness of food safety interventions in hydroponics, synthesizing their findings and evaluating their quality. We collated the extracted quantitative data from intervention studies to provide the industry and researchers with information regarding the effectiveness of interventions to mitigate human pathogens in the hydroponic produce value chain. Additionally, we identified current gaps in knowledge and provided recommendations for future research. The findings of this study can be used to guide the research community, industry, and regulators in the development of future studies and evidence-based food safety policies in hydroponics.

## 2. Materials and Methods

This study was conducted according to guidance for systematic review research methodology for food safety and food item definitions [18,19,20] and was guided by the Preferred Reporting Items for Systematic Reviews and Meta-Analyses extension for Scoping Reviews (PRISMA-ScR) checklist [21].

### 2.1. Search Strategy

A comprehensive search strategy was developed to identify the relevant literature reporting food safety interventions, risk factors, and prevalence of human pathogens in hydroponic fresh produce. A list of search terms was developed by consensus among the research team, including microbiologists, food safety specialists, plant pathologists, and experts in systematic review methodology, to retrieve peer-reviewed articles pertaining to food safety in hydroponic fresh produce. Searches were performed in five bibliographic databases: CAB Abstracts & Global Health (via Web of Science), MEDLINE (via Web of Science), Web of Science Core Collection, AGRICOLA (via EBSCOhost), and Food Science and Technology Abstracts (via EBSCOhost). The search strategy was first developed for CAB Abstracts & Global Health and tested through an iterative process with the assistance of a research librarian, using a combination of search terms for the concepts of fresh produce, hydroponics, and human pathogen. The search strategy was then adapted to the other databases using database-specific syntax. The complete search strings are shown in Supplementary Table S1. The final search update was conducted on 10 February 2025.

Search terms (n = 100 terms) pertaining to human pathogens (n = 22, e.g., *Salmonella* spp., *Listeria monocytogenes*), fresh produce (n = 71, e.g., leafy greens, lettuce, tomato), and hydroponics (n = 8, e.g., nutrient flow technique, greenhouse, controlled environment). 

### 2.2. Inclusion/Exclusion Criteria

All studies focusing on fresh produce primarily consumed raw and reporting microbial food safety outcomes, including foodborne human pathogens, surrogate spoilage organisms, and indicator microorganisms related to hydroponic crop production, were included in the review. Studies were excluded if they addressed fresh produce or other crops not consumed raw or ornamental/landscape plants. Additionally, studies concerning soil-grown produce and field production were also excluded. The review did not consider studies focusing on physical and chemical hazards, non-human disease-related organisms, or plant pathogens not associated with spoilage. Only primary research studies reporting prevalence, risk factors, and original intervention data were selected. Narrative reviews and evidence synthesis studies (e.g., scoping reviews, systematic reviews) were excluded. Only peer-reviewed journal articles were considered, while reports, conference proceedings, and other types of grey literature were excluded. Studies published in any language and from any region were eligible for inclusion.

### 2.3. Study Selection

All retrieved citations from the database searches were exported to EndNote (Clarivate, Philadelphia, PA, USA, Version 21). After removing duplicates, the remaining records were uploaded to the web-based systematic review management software Covidence (Veritas Health Innovation, Melbourne, Australia) for title/abstract screening, followed by full-text screening against the eligibility criteria. Due to time constraints, a single reviewer conducted both the title/abstract and full-text screenings. Articles that were excluded at the full-text stage were documented along with the reasons for their exclusion. All articles that passed the screening were then used in the data extraction stage.

### 2.4. Data Coding and Extraction Strategy

A predefined and pre-tested data extraction tool on ten key publications was designed in Qualtrics (Qualtrics, Provo, UT, USA) (Supplemental Table S2) and was used to extract the relevant data for this review. Data describing the study design category (prevalence, risk factor, or intervention), type of hydroponic system, type of human pathogen, produce type, and step in the hydroponic crop value chain (i.e., pre- or post-harvest, retail, etc.) were extracted from all articles. For articles reporting on food safety interventions in hydroponics, additional data were extracted (Supplemental Table S3), including intervention category (chemical, physical, or biological) and type, and the intervention treatment parameters (concentration, contact time, frequency of the application, etc.), the hydroponic system, the CEA structure, type of research facilities, types of samples collected, and the measure of the effectiveness of the intervention. The data extraction was performed by two independent reviewers, with disagreements resolved by discussion until a consensus was met. Articles published in a language other than English were translated (DeepL, Cologne, Germany) prior to data extraction. Descriptive analysis of study design categories (prevalence, risk factor, or intervention), types of hydroponic systems, types of human pathogens, produce type, and step in the hydroponic crop value chain was conducted by tabulating and summarizing the outcomes. All quantitative data were extracted in Excel (Microsoft Corporation, Redmond, WA, USA, Version 16.88), and each intervention treatment (concentration, contact time, frequency of the application, etc.) was considered to be a separate observation. The extracted intervention effectiveness data were synthesized and summarized in tables and figures. High heterogeneity of studied interventions, study designs, and data types precluded any pooled analysis of quantitative data.

### 2.5. Quality Assessment

The assessment of the methodological soundness and bias based on the elements of experimental study design included extraction of the information about availability of raw data (numerator/denominator or proportion and either numerator or denominator), measures of association/effect (OR/RR/IR/RD and its measure of variability (SE, SD, CI) or *p*-value), continuous measures (mean value, sample size, SD, and SE/Cis), the types of controls included, sample and experiment replication, appropriateness of sampling, and adequate description of methods for reproducibility. Finally, the quantitative raw or estimated data reported for each intervention were extracted and organized into categories of response (i.e., log CFU/unit, log reduction/unit, D value, etc.) for analysis.

## 3. Results and Discussion

### 3.1. Map of the Published Evidence on Food Safety in Hydroponic Fresh Produce

**Figure 2:**
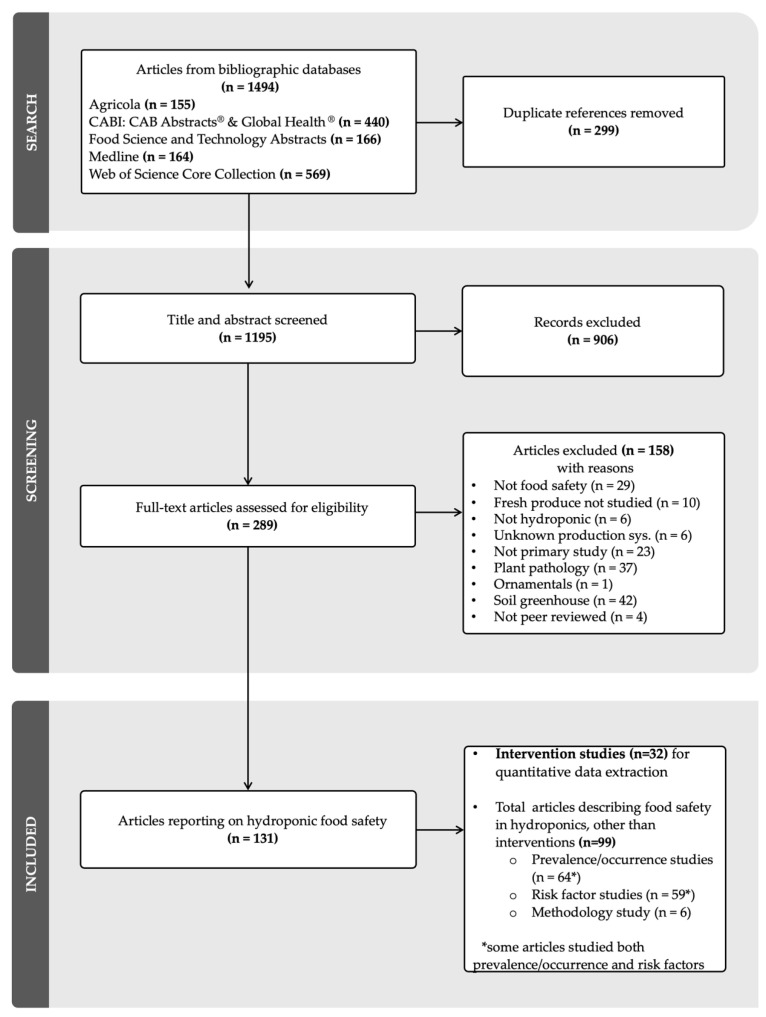
PRISMA flow diagram of the studies included in the review.

**Figure 3:**
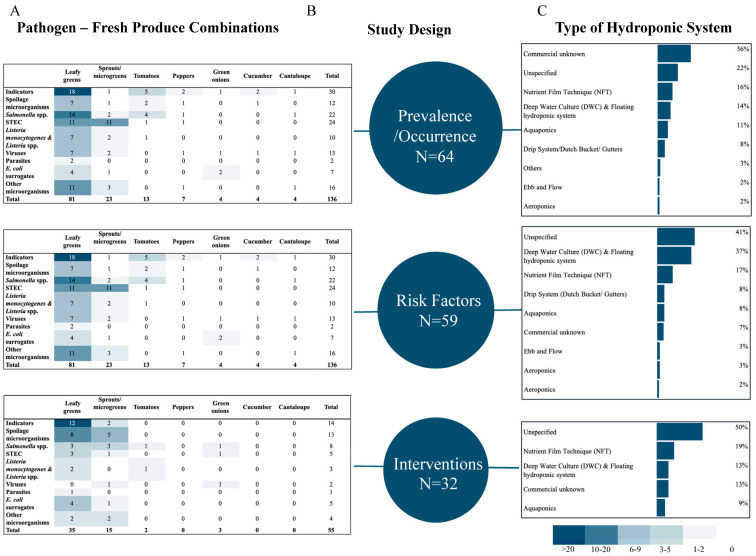
Map of the published evidence on food safety in hydroponic fresh produce showing investigated (**A**) human pathogen–produce combinations for each (**B**) study design and (**C**) hydroponic production system. Commercial unknown refers to hydroponic crops bought at retail shops and supermarkets. Unspecified includes studies that did not specify the hydroponic system used to grow crops. Viruses included norovirus and surrogates (murine norovirus, Tulane virus, canine calicivirus, and feline calicivirus), hepatitis A, rotavirus, and human adenovirus type 41.

In total, 1195 studies were identified after deduplication of the database searches. A total of 906 studies were excluded at the title/abstract screening level. The remaining 289 articles were screened at the full-text level, and 158 studies were further excluded based on predefined exclusion criteria (Figure 2). A total of 131 articles met the eligibility criteria for this review and were included in the map. The extent and content of the existing body of literature are presented in the map of included articles (n = 131), which shows studies organized by produce type, pathogen, hydroponic system, and study design (Figure 3). The full list of articles and associated journals is provided in Supplemental Data Set S1.

Most studies were published in English (84%; n = 110). Articles in Portuguese represented 11.5% (n = 15). Two studies (1.5%) each were published in Japanese and Spanish, and one study (0.8%) each was in Chinese and Korean. Articles were published in 63 different scientific journals. Over half of the articles (59.5%) were in the following journals: *Journal of Food Protection* (n = 22), *Applied and Environmental Microbiology* (n = 7), *Food Control* (n = 7), *Hygiene Alimentaria* (n = 7), *Foods* (n = 5), *Food Microbiology* (n = 4), *Journal of Applied Microbiology* (n = 4), *Acta Horticulturae* (n = 3), *Frontiers in Microbiology* (n = 3), *LWT—Food Science and Technology* (n = 3), and *Water* (n = 3). Before the year 2000, only seven articles were published, while the majority (55.7%, n = 73) were published from 2015 to February 2025. This increase in publications likely reflects the rapid growth of the hydroponic industry, heightened recognition of food safety issues, the need to better understand and manage food safety risks in hydroponic systems, and increased research funding opportunities.

Food safety-related pathogens were investigated in 64.1% (n = 84) of the 131 studies The list of investigated microorganisms is listed in Table 1, and the complete data set outlining the pathogens in each article is available in Supplemental Data Set S1. A total of 47 (35.9%) articles investigated only human pathogen indicators or surrogates. All surrogates used in hydroponic food safety investigations are summarized in Table 2. Bacterial species were investigated in 118 (90.1%) of the studies. The most commonly investigated bacterial pathogen was *Salmonella* spp. (n = 48; 36.6%), followed by Shiga toxin-producing *E. coli* (STEC) (n = 31; 23.7%), *L*. *monocytogenes* (n = 19, 14.5%), and two (1.5%) articles each included *Campylobacter* spp. and *Clostridium* spp. (Table 1). A total of 32 (24.4%) of the 131 articles studied spoilage microorganisms, including total aerobic count, yeast and mold, various coliform species, and lactic acid bacteria. Norovirus was investigated in five studies, while norovirus surrogates (murine norovirus, Tulane virus, canine calicivirus, and feline calicivirus) were studied more often (n = 10; 7.6%). Hepatitis A was investigated in four studies (3.1%), and rotavirus and human adenovirus were investigated in one study each (0.8% in each) (Table 1). Parasites were investigated in eight studies (6%), five of which studied helminths, three various non-foodborne parasites, and seven that studied fresh produce-related microparasites. With many studies reporting on indicator microorganisms or surrogates, there is a lack of empirical evidence on the behavior of human pathogenic microorganisms in the production of hydroponic fresh produce.

The published evidence indicates that pathogenic strains exhibit substantially different behavior in production systems than those of indicator microorganisms and surrogate strains [38,39]. Especially in plant systems, it is often difficult to delineate the commensal microbiome from the organisms currently used as indicators. Additionally, several pathogens that often cause foodborne illnesses in the US, such as *Campylobacter* spp., *Clostridium* spp., and toxin-producing fungi, have been rarely included in these studies. Future research studies that incorporate non-modified pathogenic strains will be of importance so that evidence-based, realistic industry recommendations can be supported. Similar recommendations have been previously published [40]. However, this can only occur if appropriate resources and biosecurity level 2 (BSL-2) grow facilities, such as research greenhouses and growth rooms, are assigned to researchers investigating food safety risks associated with fresh produce.

Leafy greens were the primary focus in most of the studies (n = 98; 74.8%), with lettuce being the most common leafy green studied in 79 papers (60.3%). Other leafy greens that were studied included spinach (n = 11), basil (n = 6), kale (n = 4), arugula (n = 2), chicory (n = 2), parsley (n = 2), and bok choy (n = 1). Forty-nine different types of sprouts and microgreens were investigated in 19 studies. Tomatoes (n= 13; 9.9%), green onions (8; 6.1%), peppers (n = 4; 3.1%), cucumber (n = 3; 2.3%), and cantaloupe (n = 1; 0.8%) were also studied.

A total of 86 of 131 (65.6%) articles investigated hydroponic crops at the production stage of the value chain. Forty-seven looked at post-harvest processing (35%); five at propagation (3.8%); and three at transport, packing, and storage (2.3%). Most articles (n = 49; 37.4%) did not specify the type of hydroponic systems used for their experiments, and 27 (20.6%) studies used commercially grown produce from unknown hydroponic systems. Among the remaining 55 studies, nutrient film technique (NFT) and deep-water culture (DWC) were the most studied (n = 21 each). Eleven studies used aquaponics, two cited using ebb and flow systems, one study each cited an indoor vertical system and NASA regenerative life support system (RLSS) test bed, and seven studies investigated crops in drip systems (Dutch buckets or gutters), three of which were commercial scale. A total of eight studies used more than one type of production system. Thirteen studies that cited DWC to grow the produce were conducted in lab-scale DWC modeling units, including small floaters, jars, styrofoam boxes, test tubes or flasks, containers, or pots to simulate DWC [23,32,34,41,42,43,44,45,46,47,48,49]. Articles describing produce–pathogen combinations that are commonly associated with outbreaks are limited. For example, while STEC and *Salmonella* spp. are responsible for most outbreaks linked to leafy greens, only 17.6% and 27.5% of the studies specifically addressed this produce–pathogen pairing, respectively. There were only two studies investigating *Campylobacter* spp., which is the second most common bacterial pathogen causing foodborne diseases in the US [50]. This is not surprising since *Campylobacter* spp. is rarely associated with fresh produce contamination. To address these gaps, it is important that future research studies use pathogen–produce combinations and production system conditions that more closely resemble commercial hydroponic production.

Based on the study design, articles were classified into four categories: risk factor (n = 59), prevalence and occurrence (n = 69), food safety interventions (n = 32), and methodology (n = 6). Risk factors such as pathogen internalization (n = 18), survival (n = 40), transfer (n = 8), and attachment and biofilms (n = 3) were investigated. Most of the prevalence and occurrence studies (n = 39) were concerned with the microbial quality of the product, but most of the experiments (n = 34) used human pathogen indicators or spoilage microorganisms rather than virulent strains of human pathogens to assess microbial quality. Of the six methodology articles, three investigated the internalization of Hepatitis A or STEC in green onions, spinach, or radish sprouts, while three investigated the detection and survival of *Salmonella* spp., STEC, or generic *E. coli* in lettuce.

### 3.2. Food Safety Interventions in Hydroponic Crop Production

Of the 131 studies in the final map, 32 investigated food safety interventions, defined as any human pathogen mitigation strategy implemented at any point in the hydroponic produce value chain. Except for three articles in Portuguese and one in Japanese, all were published in English. Articles were published in 26 different scientific journals over the past 23 years. One-half of the intervention articles (50.0%) failed to specify the hydroponic system type, and 12.5% purchased commercially grown produce originating from unknown hydroponic systems. Less than half of the articles (37.5%) explicitly stated the system type used, with one article using multiple systems. Nutrient film technique (NFT) was reported in 18.8% of studies, deep water culture (DWC) in 12.5%, and aquaponic in 9.4%. This is of concern when developing and establishing food safety intervention recommendations for implementation in commercial settings or production systems.

Quantitative data were extracted from 32 intervention studies, which included a total of 53 different interventions to prevent, control, or eliminate human pathogens in hydroponic fresh produce. The complete data set can be found in the supplement. All preharvest intervention data are summarized in Table 3 and Table 4. All intervention studies were experimental and carried out either in the laboratory or in research greenhouses. No studies were conducted on commercial operations. Four types of investigated interventions included n = 39 chemical, n = 10 physical, two multiple hurdles (chemical and physical), and two biological approaches. Overall, 22 unique chemicals were evaluated for their effectiveness against *Salmonella* spp. (n = 9), *E. coli* O157:H7 (n = 4), HAV (n = 2), human adenovirus (n = 2), *L. monocytogenes* (n = 1), *Shigella* spp. (n = 1), *S. aureus* (n = 1), *Pseudomonas aeruginosa* (n = 1), and rotavirus (n = 1). Thirteen chemicals were tested against indicators of human pathogen contamination (coliforms/*E. coli*/*Enterobacteriaceae*) and surrogates (*E. coli*, MNV, TV). Among the chemical interventions, chlorine-based sanitizers and peroxyacetic acid (PAA) were most frequently tested. This is most likely because these chemicals are commonly used in commercial hydroponic operations due to their availability and affordability. Other strategies such as calcinated calcium, Igepon^®^ TC-42, lime oil, ozone, rose bengal, and saponin were also evaluated. Physical interventions tested six unique approaches, including UV radiation, gamma-radiation, high hydrostatic pressure (HHP), dry heat seed treatment, pulse light, and dielectric barrier discharge (DPD) plasma. The effectiveness of two multiple-hurdle interventions (rose bengal/photoactivation and chlorine/UV-C) was investigated by combining physical and chemical means for the inactivation of *S.* Typhimurium at preharvest and post-harvest (Table 3 and Table 4). The two studies investigating biological interventions tested a bacteriocin-producing strain of *Pseudomonas jessenii* applied to pre- and post-harvest mung bean sprouts against *S.* Senftenberg and plant growth-promoting rhizobacteria (PGPR), which was applied individually or as a consortium, against naturally occurring *E. coli* on lettuce at the pre-harvest stage (Table 4). The limited number of studies on biological strategies presents a significant opportunity for extensive research on biological interventions unique to hydroponic environments. This is especially important because chemicals have been proven to be ineffective on multiple surfaces in hydroponics and may also exhibit detrimental effects on plant health [51,52]. Additionally, consumers may be more receptive to innovative approaches that result in chemical-free products [53].

The effectiveness of the interventions studied varied depending on the pathogen and the stage of implementation. Among the 32 articles investigating food safety interventions, 15 articles investigated a total of 28 unique preharvest mitigation approaches (Table 3 and Table 4). These fifteen articles investigated seventeen chemical, six physical, one multi-hurdle, and five biological interventions, of which *Salmonella* spp. (n = 12), indicators, and *E. coli* surrogates (n = 8) were most commonly tested. Four interventions studied *E. coli* O157:H7, five studied spoilage microorganisms or total aerobic bacteria, and one each studied *Pseudomonas aeruginosa* and *Staphylococcus aureus*. There were no reported studies on interventions against *L. monocytogenes* or norovirus in pre-harvest hydroponics. The types of preharvest interventions and their effectiveness are listed in Table 3 and Table 4. The lack of evidence regarding pre-harvest food safety strategies warrants future studies focusing on the mitigation of food safety risks in diverse hydroponic systems at this stage. This is especially important for nutrient solution treatments [11,64], DWC pond sediment removal, and mitigation of biofilms on a variety of hydroponic surfaces and substrates.

Of 17 articles that investigated post-harvest food safety strategies, 30 unique interventions were tested (Supplemental Data Set S2). The majority of the post-harvest interventions were chemical (n = 21), followed by physical (n = 7), biological (n = 1), and multi-hurdle (n = 1). Because the studies tested crops after they were harvested, the implemented food safety interventions may not necessarily be specific to hydroponically grown produce, especially since the samples were inoculated post-harvest.

The current body of evidence does not emulate the production conditions and risks specific to hydroponic crop production systems. Moreover, the systems, crops, interventions, and stage (pre- or post-harvest) in which interventions were implemented varied considerably between studies, precluding any generalization of findings or pooled analysis. Because of this, it is difficult to use the available evidence to develop food safety recommendations for the industry.

While the comparisons between the interventions were not possible due to the variability of intervention approaches and measured outcomes, the low effectiveness of chlorine-based sanitizers is apparent in several types of samples, such as NFT surfaces and on roots. While the chemical treatments generally showed some reduction of *Salmonella* on hydroponic surfaces, the elimination of the pathogen in the system remains challenging. The use of some plant growth-promoting rhizobacteria (PGPR) yielded no colonies on plates; however, enrichment was not utilized to confirm that contaminants were eliminated (Table 4). Furthermore, the authors did not provide PGPR treatment concentrations, which further hinders the utility of this intervention. Based on the published evidence, no intervention, whether chemical, physical, or biological, was able to eliminate pathogens from seeds, sprouts, or plants.

### 3.3. Quality Assessment of Intervention Studies

The assessment criteria responses are reported in Table 5. Of the 32 intervention articles, only five studies met all five criteria (raw data available, appropriate control, sample and experiment replication, and reproducibility). A total of 43.4% provided sufficient details on the methods used to reproduce the studies. One-fourth of the studies (25%) did not provide raw data, and 31.3% did not indicate whether the experiments were repeated. Although these articles were peer-reviewed prior to publication, significant details were missing. Without detailed reporting of experimental setup and methodology, the research community and stakeholders face significant barriers in validating and building upon existing research, thus complicating the translation of research findings into practical food safety measures. Future studies in hydroponic food safety will benefit from improved reporting to include specific hydroponic system types, environmental parameters, crop types, and growth stages, not only for analysis but also for policies and decision-making. Indicating the type of hydroponic system used in the studies is of particular importance because the food safety risks associated with different systems differ depending on the surface types, substrates used to support the plant, harvesting method, etc.

## 4. Conclusions

The consumption of contaminated fresh produce is responsible for over one-half of all foodborne diseases in the US and globally [65]. Therefore, for the success of controlled environment agriculture (CEA), it is critical to achieve an adequate level of food safety by implementing effective prevention and control strategies. Food safety in hydroponics has unique challenges [9,10,11]. For instance, hydroponic production is conducted in diverse growing systems that generally lack hygienic design and clean breaks and with plants that are in continuous contact with nutrition solutions and surfaces. Interventions used in the open field cannot be used in hydronic production, and therefore, any interventions should specifically address the risks in hydroponic systems and operations. In this paper, we have identified all published studies that investigated the food safety of hydroponics fresh produce. We extracted, identified, appraised, and synthesized quantitative data from the studies that investigated food safety interventions. To provide the industry with relevant recommendations on the approaches to mitigate human pathogens in hydroponic production, we constructed a list of published chemical, physical, and biological interventions (Table 3 and Table 4) that were applied pre-harvest and evaluated for their effectiveness against target microorganisms on different types of hydroponically produced crops.

However, we identified several important deficits in the available published evidence that hinder the development of food safety intervention guidelines. Of particular importance was the lack of commercial or near-commercial scale and a limited number of preharvest interventions against pathogenic strains of foodborne agents. Additionally, based on published research, the cost–benefit balance of chemical sanitizers in hydroponics may not be favorable due to the impact on yield and nutritional quality [51]. In general, chemical sanitizers have shown effectiveness in laboratory studies, but they typically achieve only a 1–2 log reduction of human pathogens on produce [17]. In hydroponics, their effectiveness is further limited by their negative impact on plant health [51]. Therefore, it is essential to look beyond chemical solutions and explore novel biological approaches and new technologies to mitigate food safety risks in hydroponics.

Leafy greens, and in particular lettuce, were the focus in most of the reviewed studies. Although this is understandable given the short life cycle of lettuce, other crops with longer growth cycles that are commonly grown in hydroponics (e.g., strawberries, tomatoes, peppers, cucumbers, etc.) have been neglected, and validated approaches to mitigate human pathogens in these systems are lacking. Because food safety risks in these systems substantially differ from the risks in leafy green production, it is not possible to make inferences about interventions and their effectiveness in other crops based on leafy green research. Future research should focus on a broader spectrum of crops in hydroponic systems.

The quality appraisal of current evidence revealed a significant lack of details in reporting methods and intervention outcomes. To maximize the benefits of future research studies and ensure their effective translation into practice, policy, and guidelines, we recommend that future articles explicitly state the hydroponic system used in the study and provide a detailed description of the crop production methods employed. Additionally, researchers should include raw data in all future articles and reports investigating food safety interventions in hydroponics.

We have provided a table (Table 2) summarizing the surrogates used to assess the food safety interventions in hydroponics. While surrogates and indicator microorganisms are more convenient for testing due to the lowered biosafety level, and studies using them provide valuable information, they have been reported to respond differently to food safety interventions [38,39]. Studies that use pathogenic strains will allow for more reliable recommendations in hydroponic systems because they more closely model their survival and colonization in the plant system.

Because hydroponic production holds the potential to enhance human health and nutrition and promote sustainable agricultural practices, it is critical that food safety be a priority in research and practice. The existing evidence reviewed in this study and the identified gaps in research reflect the complexity of conducting scalable, reproducible experiments that will result in data-driven food safety intervention recommendations that are applicable to diverse cropping systems in hydroponics. The state of evidence shows that we are still a long way from developing and making practical recommendations. Food safety research in hydroponic food production is still in the infancy stage. To advance the field, considerably more resources that go beyond laboratory facilities, including indoor growing spaces with adequate biosafety clearance, are necessary. Establishing a food safety culture and implementing validated food safety interventions can ensure a safe product and a thriving hydroponic industry.

## Figures and Tables

**Table 1:** Types of microorganisms investigated in the reviewed studies describing food safety in hydroponic fresh produce.

Microorganism	No. Studies *	Percent (%)
***Bacteria***		
*Campylobacter* spp.	2	1.5
*Clostridium* spp.	2	1.5
*Listeria monocytogenes*	19	14.5
*Listeria* spp.	4	3.1
*Salmonella* spp.	48	36.6
Shiga toxin-producing *E. coli* (STEC)	31	23.7
*E. coli* surrogates	7	5.3
Indicator microorganisms (*E. coli*/coliforms, Enterobacteriaceae)	60	45.8
Total aerobic bacteria	25	19.1
Spoilage bacteria (mesophilic, psychotropic, lactic acid)	4	3.1
Other bacteria	23	17.6
***Viruses***		
Norovirus	5	3.8
Norovirus surrogates	10	7.8
Hepatitis A	4	3.1
Human adenovirus	1	0.8
Rotavirus	1	0.8
***Parasites***		
*Giardia lamblia*	2	1.5
*Cryptosporidium* spp.	2	1.5
*Toxoplasma gondii*	1	0.8
Other parasites	6	4.6
***Yeast and Molds***	17	13.0

* Data were extracted from 131 studies (N = 131) with some studies investigating multiple microorganisms.

**Table 2:** Human pathogen surrogates investigated in the reviewed studies describing food safety in hydroponic fresh produce.

Surrogate	Surrogate Designation	Reference
*E. coli*	*E. coli* KM1	[22,23]
	*E. coli* KSC1	[24]
	Bioluminescent *E. coli* P36	[25]
	*E. coli* ATCC 25922, *E. coli* ATCC 10798 (*E. coli* K12)	[17]
	*E. coli* ATCC 35218TM	[26]
	*E. coli P4, E. coli P13, and E. coli P68*	[27]
Norovirus	Murine norovirus	[28,29,30,31,32]
	Feline Calicivirus	[33]
	Tulane Virus	[28,34,35,36]
	Canine Calicivirus 48	[37]

**Table 3:** Chemical and multi-hurdle preharvest intervention parameters and reported effectiveness in mitigating microbial contamination in hydroponic fresh produce systems.

Intervention Parameters			Crop	Sample Type	Organism	Quantitative Response			System	Reference
Treatment	Concentration	Time				Control ± SD ^a^	Treatment ± SD ^a^	Units		
Calcinated calcium	0.10%	12 h	Sprouts—radish	Plant	*E. coli* O157:H7	3.60 ± 0.60 × 10^7^	1.00 ± 0.50 × 10^3^	CFU/mL	NR ^d^	[54]
	0.20%						420 ± 30.00	CFU/mL		
	0.30%						50.00 ± 5.00	CFU/mL		
	0.35%						5.00 ± 0.60	CFU/mL		
	0.40%						<3.00	CFU/mL		
	0.50%						<3.00	CFU/mL		
	1.00%						<3.00	CFU/mL		
Igepon TC-42	240 ppm	59 days	Sprouts—wheat	Roots	*Pseudomonas aeruginosa*	~10^4^–10^6 b^	~10^3^–10^4 b^	CFU/g dry	NFT	[24]
				Nutrient solution			~10^2 b^	CFU/mL		
				PVC surface			~10^2 b^	CFU/cm^2^		
				Roots	*E. coli* generic	~10^4 b^	BDL	CFU/g dry		
				Nutrient solution			BDL	CFU/mL		
				PVC surface			BDL	CFU/cm^2^		
				Roots	*Staphylococcus aureus*	~10^3^–10^4 b^	BDL	CFU/g dry		
				Nutrient solution			BDL	CFU/mL		
				PVC Surface			BDL	CFU/cm^2^		
	240 ppm	27 days	Lettuce	Roots	*Pseudomonas aeruginosa*	~10^5 b^	~10^3^–10^7 b^	CFU/g dry		
				Nutrient solution			~10^2^–10^3 b^	CFU/mL		
				PVC surface			~10^2^–10^3 b^	CFU/cm^2^		
				Roots	*E. coli* generic	~10^3^–10^4 b^	BDL ^e^	CFU/g dry		
				Nutrient solution			BDL	CFU/mL		
				PVC surface			BDL	CFU/cm^2^		
				Roots	*Staphylococcus aureus*	~10^3^–10^4 b^	BDL	CFU/g dry		
				Nutrient solution				CFU/mL		
				PVC surface				CFU/cm^2^		
Ozonated water	0.5 mg/L	3 min	Chard	Nutrient solution	*E. coli* ATCC 35218TM	5.3 ^b^	5.8 ^b^	Log CFU/mL	DWC	[26]
				Roots		4.87	4.41	Log CFU/g		
				Leaves		4.81	4.32	Log CFU/g		
	2 mg/L	3 min		Nutrient solution		5.3 ^b^	5.0 ^b^	Log CFU/mL		
				Roots		4.87	5.21	Log CFU/g		
				Leaves		4.81	4.34	Log CFU/g		
Ozonated water	5.8 mg/L	3 doses	Japanese mustard spinach	Nutrient solution	Coliforms	2.5 ^b^	2.5 ^b^	Log CFU/mL	NFT	[55] ^a^
				Leaves		3.5 ^b^	3.8 ^b^	Log CFU/g		
				Nutrient solution	TAC	5.2 ^b^	4.8 ^b^	Log CFU/mL		
				Leaves		4.0 ^b^	5.0 ^b^	Log CFU/g		
Sodium chloride	150 mM		Sea fennel	Plant	Psychrophilic bacteria	5.81 ± 0.28	5.25 ± 0.45	Log CFU/g	DWC	[56]
					Mesophilic bacteria	5.40 ± 0.31	5.24 ± 0.36			
					Enterobacteria	5.15 ± 0.38	3.90 ± 0.98			
					Yeast and mold	3.89 ± 0.18	3.34 ± 0.12			
Citric acid	1.5 mM	14 days	Lettuce	Nutrient solution	*E. coli* generic	3.3 ± 0.10	0	Log CFU/mL	NR	[57]
				Roots		0 ± 0.00	0	Log CFU/g		
				Leaves		3.2 ± 0.10	0	Log CFU/g		
	2.5 mM			Nutrient solution		3.3 ± 0.10	0	Log CFU/mL		
				Roots		0 ± 0.00	0	Log CFU/g		
				Leaves		3.2 ± 0.10	0	Log CFU/g		
Saponin	12.5 µg/mL	35 days	Lettuce	Nutrient solution	*E. coli* surrogate cocktail	0.89	2.32	Log CFU/mL +1	NFT	[27]
	25 µg/mL					0.89	2.15	Log CFU/mL +1		
	50 µg/mL					0.89	3.21	Log CFU/mL +1		
	100 µg/mL					0.89	4.61	Log CFU/mL +1		
Sodium hypochlorite	100 ppm	1 h	Lettuce	Reservoir ABS plastic	*Salmonella* Typhimurium	0.45 ± 0.19	0.68 ± 0.04	Log reduction	NFT	[51]
				Top-cover UV-stabilized PVC		0.62 ± 0.10	1.83 ± 0.43	Log reduction		
				Channel UV-stabilized PVC		1.53 ± 0.27	0.68 ± 0.08	Log reduction		
				Drain line PVC		2.36 ± 0.60	0.86 ± 0.43	Log reduction		
	200 ppm			Reservoir ABS		0.45 ± 0.19	1.78 ± 0.32	Log reduction		
				Top-cover UV-stabilized PVC		0.62 ± 0.10	3.21 ± 0.4	Log reduction		
				Channel UV-stabilized PVC		1.53 ± 0.27	3.42 ± 0.19	Log reduction		
				Drain line PVC		2.36 ± 0.60	0.89 ± 0.07	Log reduction		
Sodium hypochlorite	100 ppm	3 h	Lettuce	Reservoir ABS plastic	*Salmonella* Typhimurium	4.49 ± 0.07	3.90 ± 1.18	Log reduction	NFT	[51]
				Top-cover UV-stabilized PVC		4.28 ± 0.34	3.65 ± 0.61	Log reduction		
				Channel UV-stabilized PVC		3.68 ± 0.35	4.22 ± 0.54	Log reduction		
				Drain line PVC		4.90 ± 0.16	4.98 ± 0.14	Log reduction		
	200 ppm			Reservoir ABS		4.49 ± 0.07	4.61 ± 0.33	Log reduction		
				Top-cover UV-stabilized PVC		4.28 ± 0.34	4.67 ± 0.30	Log reduction		
				Channel UV-stabilized PVC		3.68 ± 0.35	5.07 ± 0.08	Log reduction		
				Drain line PVC		4.90 ± 0.16	5.20 ± 0.00	Log reduction		
Sodium hypochlorite	5 ppm		Lettuce	Roots	*E. coli* O157:H7	1.0 × 10^2^	1.67 × 10^2^	CFU/g	Ebb and Flow	[58] ^c^
				Urethane substrate		8.0 × 10^2^	3.67 × 10^2^	CFU/mL		
				Roots	*TAC*	1.87 × 10^2^	9.67 × 10^2^	CFU/g		
				Urethane substrate		9.67 × 10^2^	4.0 × 10^2^	CFU/mL		
Chlorine dioxide	10 ppm	1 h	Lettuce	Reservoir ABS	*Salmonella*	0.45 ± 0.19	0.57 ± 0.07	Log reduction	NFT	[51]
					Typhimurium					
				Top-cover UV-stabilized PVC		0.62 ± 0.10	1.35 ± 0.23	Log reduction		
				Channel UV-stabilized PVC		1.53 ± 0.27	1.37 ± 0.51	Log reduction		
				Drain line PVC		2.36 ± 0.60	1.21 ± 0.21	Log reduction		
	50 ppm			Reservoir ABS		0.45 ± 0.19	0.67 ± 0.10	Log reduction		
				Top-cover UV-stabilized PVC		0.62 ± 0.10	4.14 ± 0.40	Log reduction		
				Channel UV-stabilized PVC		1.53 ± 0.27	2.39 ± 0.29	Log reduction		
				Drain line PVC		2.36 ± 0.60	2.46 ± 0.06	Log reduction		
Chlorine dioxide	10 ppm	3 h	Lettuce	Reservoir ABS	*Salmonella*	4.49 ± 0.07	5.08	Log reduction		
					Typhimurium					
				Top-cover UV-stabilized PVC		4.28 ± 0.34	3.90 ± 0.32	Log reduction		
				Channel UV-stabilized PVC		3.68 ± 0.35	4.17 ± 0.28	Log reduction		
				Drain line PVC		4.90 ± 0.16	4.87 ± 0.23	Log reduction		
	50 ppm			Reservoir ABS		4.49 ± 0.07	5.34 ± 0.26	Log reduction		
				Top-cover UV-stabilized PVC		4.28 ± 0.34	4.13 ± 0.03	Log reduction		
				Channel UV-stabilized PVC		3.68 ± 0.35	4.83 ± 0.23	Log reduction		
				Drain line PVC		4.90 ± 0.16	5.11 ± 0.09	Log reduction		
SaniDate^®^ 12.0	100 ppm	1 h	Lettuce	Reservoir ABS	*Salmonella*	0.45 ± 0.19	5.60 ± 0.00	Log reduction	NFT	[51]
					Typhimurium					
				Top-cover UV-stabilized PVC		0.62 ± 0.10	5.18 ± 0.00	Log reduction		
				Channel UV-stabilized PVC		1.53 ± 0.27	4.49 ± 0.35	Log reduction		
				Drain line PVC		2.36 ± 0.60	4.24 ± 0.51	Log reduction		
	200 ppm			Reservoir ABS		0.45 ± 0.19	5.60 ± 0.00	Log reduction		
				Top-cover UV-stabilized PVC		0.62 ± 0.10	5.18 ± 0.00	Log reduction		
				Channel UV-stabilized PVC		1.53 ± 0.27	5.15 ± 0.00	Log reduction		
				Drain line PVC		2.36 ± 0.60	5.21 ± 0.00	Log reduction		
SaniDate^®^ 12.0	100 ppm	3 h	Lettuce	Reservoir ABS	*Salmonella*	4.49 ± 0.07	5.60 ± 0.00	Log reduction		
					Typhimurium					
				Top-cover UV-stabilized PVC		4.28 ± 0.34	5.18 ± 0.00	Log reduction		
				Channel UV-stabilized PVC		3.68 ± 0.35	5.15 ± 0.00	Log reduction		
				Drain line PVC		4.90 ± 0.16	5.03 ± 0.10	Log reduction		
	200 ppm			Reservoir ABS		4.49 ± 0.07	5.60 ± 0.00	Log reduction		
				Top-cover UV-stabilized PVC		4.28 ± 0.34	5.18 ± 0.00	Log reduction		
				Channel UV-stabilized PVC		3.68 ± 0.35	5.15 ± 0.00	Log reduction		
				Drain line PVC		4.90 ± 0.16	5.20 ± 0.00	Log reduction		
Green Shield^®^	5%	1 h	Lettuce	Reservoir ABS	*Salmonella*	0.45 ± 0.19	5.60 ± 0.00	Log reduction	NFT	[51]
					Typhimurium					
				Top-cover UV-stabilized PVC		0.62 ± 0.10	5.18 ± 0.00	Log reduction		
				Channel UV-stabilized PVC		1.53 ± 0.27	5.15 ± 0.00	Log reduction		
				Drain line PVC		2.36 ± 0.60	5.21 ± 0.00	Log reduction		
Green Shield^®^	5%	3 h	Lettuce	Reservoir ABS	*Salmonella*	4.49 ± 0.07	5.60 ± 0.00	Log reduction		
					Typhimurium					
				Top-cover UV-stabilized PVC		4.28 ± 0.34	5.18 ± 0.00	Log reduction		
				Channel UV-stabilized PVC		3.68 ± 0.35	5.15 ± 0.00	Log reduction		
				Drain line PVC		4.90 ± 0.16	5.20 ± 0.00	Log reduction		
PACE Kleen Grow™	2%	1 h	Lettuce	Reservoir ABS	*Salmonella*	0.45 ± 0.19	5.60 ± 0.00	Log reduction	NFT	[51]
					Typhimurium					
				Top-cover UV-stabilized PVC		0.62 ± 0.10	5.18 ± 0.00	Log reduction		
				Channel UV-stabilized PVC		1.53 ± 0.27	5.15 ± 0.00	Log reduction		
				Drain line PVC		2.36 ± 0.60	5.21 ± 0.00	Log reduction		
PACE Kleen Grow™	2%	3 h	Lettuce	Reservoir ABS	*Salmonella*	4.49 ± 0.07	5.60 ± 0.00	Log reduction		
					Typhimurium					
				Top-cover UV-stabilized PVC		4.28 ± 0.34	5.18 ± 0.00	Log reduction		
				Channel UV-stabilized PVC		3.68 ± 0.35	5.15 ± 0.00	Log reduction		
				Drain line PVC		4.90 ± 0.16	5.20 ± 0.00	Log reduction		
Virkon^®^	1%	1 h	Lettuce	Reservoir ABS	*Salmonella*	4.49 ± 0.07	5.60 ± 0.00	Log reduction	NFT	[51]
					Typhimurium					
				Top-cover UV-stabilized PVC		4.28 ± 0.34	5.18 ± 0.00	Log reduction		
				Channel UV-stabilized PVC		3.68 ± 0.35	5.15 ± 0.00	Log reduction		
				Drain line PVC		4.90 ± 0.16	5.21 ± 0.00	Log reduction		
Virkon^®^	1%	3 h	Lettuce	Reservoir ABS	*Salmonella*	4.49 ± 0.07	5.60 ± 0.00	Log reduction		
					Typhimurium					
				Top-cover UV-stabilized PVC		4.28 ± 0.34	5.18 ± 0.00	Log reduction		
				Channel UV-stabilized PVC		3.68 ± 0.35	5.15 ± 0.00	Log reduction		
				Drain line PVC		4.90 ± 0.16	5.20 ± 0.00	Log reduction		
Zerotol^®^	5%	1 h	Lettuce	Reservoir ABS	*Salmonella*	4.49 ± 0.07	5.60 ± 0.00	Log reduction	NFT	[51]
					Typhimurium					
				Top-cover UV-stabilized PVC		4.28 ± 0.34	5.18 ± 0.00	Log reduction		
				Channel UV-stabilized PVC		3.68 ± 0.35	5.15 ± 0.00	Log reduction		
				Drain line PVC		4.90 ± 0.16	5.21 ± 0.00	Log reduction		
Zerotol^®^	5%	3 h	Lettuce	Reservoir ABS	*Salmonella*	4.49 ± 0.07	5.60 ± 0.00	Log reduction		
					Typhimurium					
				Top-cover UV-stabilized PVC		4.28 ± 0.34	5.18 ± 0.00	Log reduction		
				Channel UV-stabilized PVC		3.68 ± 0.35	5.15 ± 0.00	Log reduction		
				Drain line PVC		4.90 ± 0.16	5.20 ± 0.00	Log reduction		
Rose Bengal	100 µmol/L	30 min	Lettuce	Seedlings	*Salmonella*	5.88 ± 0.47	0.94 ± 0.42	Log reduction/seedling	NR	[59]
					Typhimurium					
Rose Bengal + PDI ^f^	100 µmol/L and 180 W	30 min	Lettuce	Seedlings	*Salmonella*	5.88 ± 0.47	2.77 ± 0.49	Log CFU/seedling	NR	[59]
					Typhimurium					

^a^ SD—Standard deviation when reported in the articles. ^b^ Data were extrapolated from the article by the authors. ^c^ Article translated to English using DeepL (www.deepl.com/en/translator accessed on 12 December 2024). ^d^ NP-Not reported in the article. ^e^ BDL—Below the detection limit of the assay. ^f^ PDI—Photodynamic inactivation.

**Table 4:** Physical and biological preharvest intervention parameters and reported effectiveness in mitigating microbial contamination in hydroponic fresh produce systems.

Intervention Parameters			Crop	Sample Type	Organism	Quantitative Response			System	Reference
Treatment	Concentration	Time				Control ± SD ^a^	Treatment ± SD ^a^	Units		
UV	30–80 mJ/cm2 @ 170–26 L/min; 80 W	6 weeks	Lettuce	Nutrient solution	*E. coli* generic	4.30 ± 0.40	0.80 ± 0.30	CFU/L	Aquaponic	[48]
					Coliforms	4.50 ± 0.40	1.60 ± 0.50	CFU/L		
UV	900 lm/432.6 W·s·m^−2^; 15 W	118 days	Lettuce	Plant	TAC	2.87	3.58	Log CFU/g	Aquaponic; DWC	[60]
				Nutrient solution		4.05	4.33	Log CFU/mL		
			Basil	Plant		4.89	4.69	Log CFU/g		
				Nutrient solution		4.05	4.33	Log CFU/mL		
			Lettuce	Plant	Coliforms	0.12	0.95	Log CFU/g		
				Nutrient solution		2.21	1.68	Log CFU/mL		
			Basil	Plant		1.68	1.79	Log CFU/g		
				Nutrient solution		2.21	1.68	Log CFU/mL		
UV-C 254 nm	950 mJ/cm^2^ 1.583 mW/cm^2^, 15 W	10 min/6 doses	Sprouts—mung bean	Nutrient solution	*Salmonella* Typhimurium	3.88 ± 0.31	2.98 ± 0.62	Log CFU/mL	NR ^b^	[61]
DBD plasma water	0.3 ppm of O3; 3.2 μM OH; 4.6 μM H_2_O_2_; 150 μM (NO_x_)	5 min	Sprouts—soybean	Nutrient solution	TAC	NA	4.30	Log reduction	NR	[62]
		2 min			*Salmonella* Typhimurium	NA	7.00	Log reduction		
Heat treatment (dry)	80 °C/85 °C	3/5 days	Lettuce	Seeds	*E. coli* O157:H7	Positive	Negative		NR	[62]
Heat treatment (steam)	80 °C	10 min	Lettuce	Styrofoam	*E. coli* O157:H7	Positive	Positive		NR	[62]
	80 °C	20/30/60 min				Positive	Negative			
	121 °C	10/20 min				Positive	Negative			
*Pseudomonas Jessenii* LTH 5930	10^8^ cfu/g	after inoculation, 12 h	Sprouts—mung bean	Seeds	*Salmonella*	~10^7^	~10^5^	CFU/g	NR	[63]
					Senftenberg					
		before inoculation, 12 h	Sprouts—mung bean	Seeds		~10^8^	<10^1^	CFU/g		
*Streptomyces* sp. KACC 21110	NR	14 days	Lettuce	Nutrient solution	*E. coli* generic	3.3 ± 0.10	2.3 ± 0.10	Log CFU/mL	NR	[57]
				Roots		0 ± 0.00	1.6 ± 0.10	Log CFU/g		
				Leaves		3.2 ± 0.10	2.6 ± 0.10	Log CFU/g		
*Bacillus velezensis* KACC 14540	NR	14 days		Nutrient solution	*E. coli* generic	3.3 ± 0.10	0 ± 0.00	Log CFU/mL		
				Roots		0 ± 0.00	0 ± 0.00	Log CFU/g		
				Leaves		3.2 ± 0.10	1.8 ± 0.10	Log CFU/g		
*Bacillus velezensis* KACC 14542	NR	14 days		Nutrient solution	*E. coli* generic	3.3 ± 0.10	0 ± 0.00	Log CFU/mL		
				Roots		0 ± 0.00	0 ± 0.00	Log CFU/g		
				Leaves		3.2 ± 0.10	2.1 ± 0.10	Log CFU/g		
PGPR ^c^ all isolates mixed	NR	14 days		Nutrient solution	*E. coli* generic	3.3 ± 0.10	2.1 ± 0.10	Log CFU/mL		
				Roots		0 ± 0.00	1.1 ± 0.10	Log CFU/g		
				Leaves		3.2 ± 0.10	1.9 ± 0.10	Log CFU/g		

^a^ SD—Standard deviation when reported in the articles. ^b^ Not reported in the article. ^c^ PGPR—Plant growth-promoting rhizobacteria.

**Table 5:** Characteristics of pre- and post-harvest intervention studies (n = 32) describing the effectiveness of food safety intervention in hydroponic production.

Quality Criteria ^a^	Number of Intervention Studies (%)
**Raw data**	
No	3 (9.4)
Some raw data ^b^	5 (15.6)
Yes	28 (87.5)
Mean ± standard deviation	15 (46.9)
Mean ± standard error	4 (12.5)
Mean	15 (46.9)
D-value ± standard deviation	1 (3.1)
Incidence (no. positive sample/total number of samples)	1 (3.1)
**Control type**	
Non-treated	27 (84.4)
Non-inoculated	4 (12.5)
Not reported	1 (3.1)
**Technical replication**	
Triplicate or more	22 (68.8)
Duplicate	6 (21.9)
Single	1 (3.1)
Not reported	3 (9.4)
**Experiment replication**	
Triplicate or more	15 (46.9)
Duplicate	7 (21.9)
Not reported	10 (31.3)
**Methodology**	
Sufficient detail to reproduce the study	11 (43.4)
Insufficient details	21 (65.6)

^a^ Definitions of quality criteria are provided in the quality assessment and data extraction tool (Supplemental Table S3). ^b^ Studies reported raw data only for some tested intervention outcomes.

## Data Availability

The original contributions presented in this study are included in the article/supplementary material. Further inquiries can be directed to the corresponding authors.

## Supplementary Materials

The following supporting information can be downloaded at https://www.mdpi.com/article/10.3390/foods14132308/s1. Table S1. Search queries for bibliographic databases: A. CAB Abstracts & Global Health, B. MEDLINE (via Web of Science), C. Web of Science Core Collection, D. AGRICOLA (via EBSCOhost), and E. Food Science and Technology Abstracts (via EBSCOhost); Table S2. Criteria and definitions for relevance screening (RS1 and RS2) in identifying and characterizing articles on hydroponic food safety; Table S3. Data extraction and quality assessment tool to map intervention studies related to hydroponic production. Supplemental Data Set S1: Map of all 131 relevant studies included in this study; Supplemental Data Set S2: Map of all 32 intervention studies.

## Abbreviations

The following abbreviations are used in this manuscript:

BDL	Below the detection limit
CEA	Controlled environment agriculture
DBD	Dielectric barrier discharge
DWC	Deep water culture
*E. coli*	*Escherichia coli*
HAV	Hepatitis A virus
MNV	*Murine norovirus*
NR	Not reported
PDI	Photodynamic inactivation
PGPR	Plant growth-promoting rhizobacteria
RT	Rotavirus
SD	Standard deviation
STEC	Shiga toxin-producing *Escherichia coli*
TAC	Total aerobic count
TV	Tulane virus
