# Supplementary material for: Food Safety in Hydroponic Food Crop Production: A Review of Intervention Studies to Control Human Pathogens
Source: Foods. 2025 Jun 29;14(13):2308. doi: 10.3390/foods14132308 (PMC12248475; doi:10.3390/foods14132308)
Supplement: Supplementary file 1 [file foods-14-02308-s001.zip › Figure 3_revised.pptx]

## Slide 1
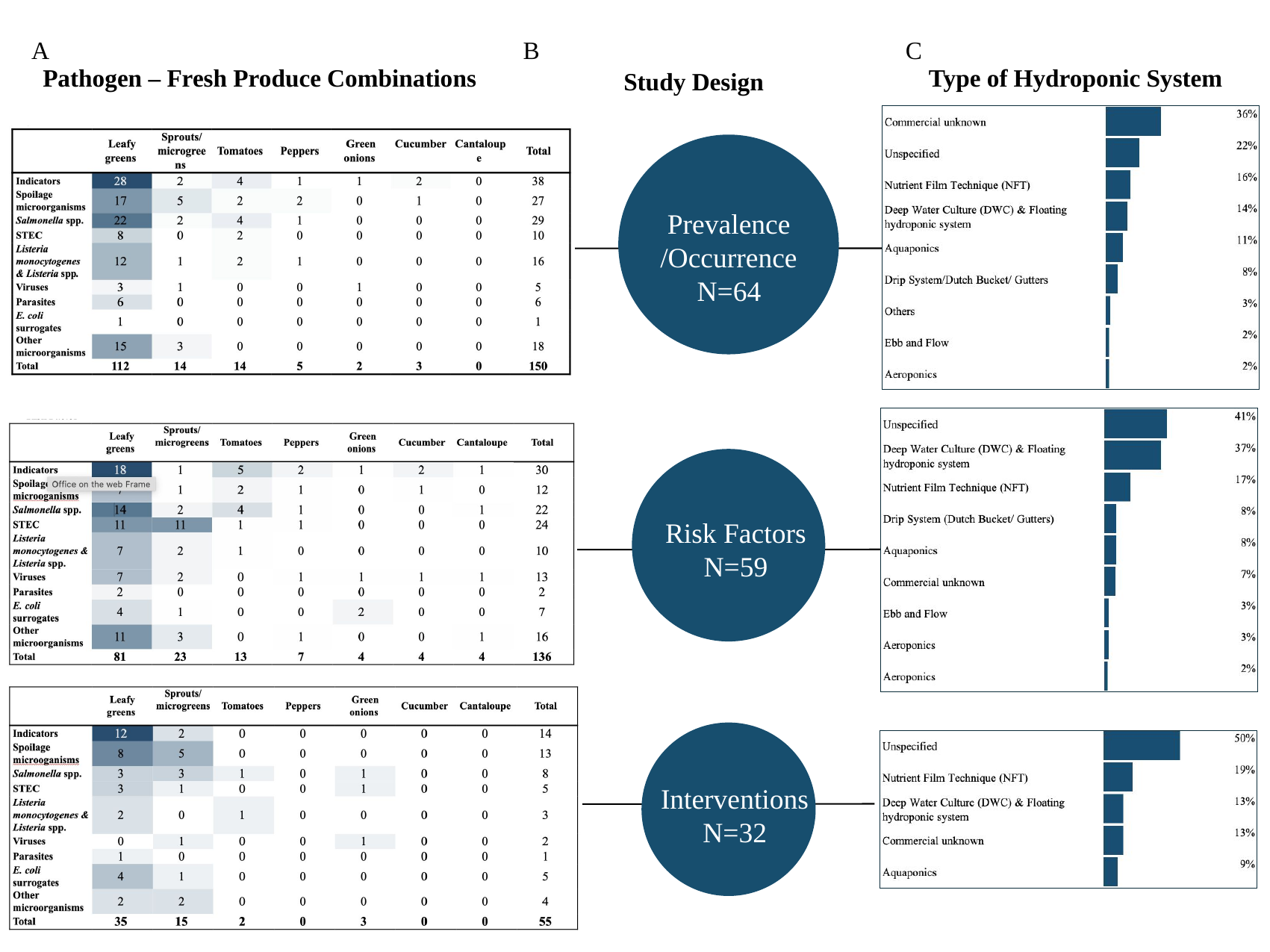

A
B
C
Type of Hydroponic System
Pathogen – Fresh Produce Combinations
Study Design
Prevalence
/Occurrence
N=64
Risk Factors
N=59
Interventions
N=32
>20
1-2
6-9
10-20
0
3-5
