# Supplementary material for: Food Safety in Hydroponic Food Crop Production: A Review of Intervention Studies to Control Human Pathogens
Source: Foods. 2025 Jun 29;14(13):2308. doi: 10.3390/foods14132308 (PMC12248475; doi:10.3390/foods14132308)
Supplement: Supplementary file 1 [file foods-14-02308-s001.zip › Supplementary_Table_Review.pdf]

Table S1. Search queries for bibliographic databases

A. CAB Abstracts & Global Health

Block	Terms
Human pathogen	TS=(campylobacter OR clostridium OR coliform* OR cryptosporidium OR cyclospora OR e.coli OR "e. coli" OR enterobacter* OR escherichia OR giardia OR hepatitis OR listeria OR non-o157 OR norovirus* OR norwalk OR o157 OR salmonella OR shigella OR staphylococcus OR stec OR toxoplasma OR vibrio OR yersinia)<br><br>AND
Fresh produce	TS=(vegetable* OR produce OR leafy OR green* OR microgreen* OR micro-green* OR babygreen* OR "spring mix" OR salad* OR herb* OR sprout* OR artichoke OR arugula OR basil OR berries OR berry OR blackber* OR blueber* OR "bok choy" OR brassica* OR broccoli OR cabbage* OR cantaloupe* OR carrot* OR cauliflower OR celer* OR chard OR chervil OR chicory OR cilantro OR collard* OR coriander OR cress OR cucumber* OR current* OR dill OR endive OR garlic OR gourd OR honeydew* OR kale OR kohlrabi* OR leek* OR lettuce* OR melon* OR mesclun OR mint OR mizuna OR mulber* OR mustard OR onion* OR oregano OR "pak choi" OR parsley OR pepper* OR radicchio OR radish OR raspber* OR rocket OR savory OR shallot* OR spinach OR sprout* OR squash* OR strawber* OR tomato* OR turmeric* OR turnip* OR watercress OR watermelon* OR zucchini*)<br><br>AND
Hydroponics	TS=(aeroponic* OR aquaponic* OR "controlled environment" OR hydroponic* OR "nutrient film technique*" OR "nutrient solution*" OR soilless OR soil-free)

B. MEDLINE (via Web of Science)

Block	Terms
Human pathogen	TS=(campylobacter OR clostridium OR coliform* OR cryptosporidium OR cyclospora OR e.coli OR "e. coli" OR enterobacter* OR escherichia OR giardia OR hepatitis OR listeria OR non-o157 OR norovirus* OR norwalk OR o157 OR salmonella OR shigella OR staphylococcus OR stec OR toxoplasma OR vibrio OR yersinia) OR MHX=(campylobacter OR clostridium OR cryptosporidium OR cyclospora OR enterobacteriaceae OR escherichia OR giardia OR hepatitis OR listeria OR norovirus OR shigella OR salmonella OR staphylococcus OR toxoplasma OR vibrio OR yersinia)<br><br>AND

Fresh produce	TS=(vegetable* OR produce OR leafy OR green* OR microgreen* OR micro-green* OR babygreen* OR "spring mix" OR salad* OR herb* OR sprout* OR artichoke OR arugula OR basil OR berries OR berry OR blackber* OR blueber* OR "bok choy" OR brassica* OR broccoli OR cabbage* OR cantaloupe* OR carrot* OR cauliflower OR celer* OR chard OR chervil OR chicory OR cilantro OR collard* OR coriander OR cress OR cucumber* OR current* OR dill OR endive OR garlic OR gourd OR honeydew* OR kale OR kohlrabi* OR leek* OR lettuce* OR melon* OR mesclun OR mint OR mizuna OR mulber* OR mustard OR onion* OR oregano OR "pak choi" OR parsley OR pepper* OR radicchio OR radish OR raspber* OR rocket OR savory OR shallot* OR spinach OR sprout* OR squash* OR strawber* OR tomato* OR turmeric* OR turnip* OR watercress OR watermelon* OR zucchini*) OR MHX=("crops, agricultural" OR fruit OR "pants, edible" OR "raw foods" OR vegetables)
	AND
Hydroponics	TS=(aeroponic* OR aquaponic* OR "controlled environment" OR hydroponic* OR "nutrient film technique*" OR "nutrient solution*" OR soilless OR soil-free)

C. Web of Science Core Collection

Block	Terms
Human pathogen	TS=(campylobacter OR clostridium OR coliform* OR cryptosporidium OR cyclospora OR e.coli OR "e. coli" OR enterobacter* OR escherichia OR giardia OR hepatitis OR listeria OR non-o157 OR norovirus* OR norwalk OR o157 OR salmonella OR shigella OR staphylococcus OR stec OR toxoplasma OR vibrio OR yersinia)
	AND
Fresh produce	TS=(vegetable* OR produce OR leafy OR green* OR microgreen* OR micro-green* OR babygreen* OR "spring mix" OR salad* OR herb* OR sprout* OR artichoke OR arugula OR basil OR berries OR berry OR blackber* OR blueber* OR "bok choy" OR brassica* OR broccoli OR cabbage* OR cantaloupe* OR carrot* OR cauliflower OR celer* OR chard OR chervil OR chicory OR cilantro OR collard* OR coriander OR cress OR cucumber* OR current* OR dill OR endive OR garlic OR gourd OR honeydew* OR kale OR kohlrabi* OR leek* OR lettuce* OR melon* OR mesclun OR mint OR mizuna OR mulber* OR mustard OR onion* OR oregano OR "pak choi" OR parsley OR pepper* OR radicchio OR radish OR raspber* OR rocket OR savory OR shallot* OR spinach OR sprout* OR squash* OR strawber* OR tomato* OR turmeric* OR turnip* OR watercress OR watermelon* OR zucchini*)
	AND
Hydroponics	TS=(aeroponic* OR aquaponic* OR "controlled environment" OR hydroponic* OR "nutrient film technique*" OR "nutrient solution*" OR soilless OR soil-free)

D. AGRICOLA (via EBSCOhost)

Block	Terms
Human pathogen	TS=(campylobacter OR clostridium OR coliform* OR cryptosporidium OR cyclospora OR e.coli OR "e. coli" OR enterobacter* OR escherichia OR giardia OR hepatitis OR listeria OR non-o157 OR norovirus* OR norwalk OR o157 OR salmonella OR shigella OR staphylococcus OR stec OR toxoplasma OR vibrio OR yersinia)
	AND
Fresh produce	TS=(vegetable* OR produce OR leafy OR green* OR microgreen* OR micro-green* OR babygreen* OR "spring mix" OR salad* OR herb* OR sprout* OR artichoke OR arugula OR basil OR berries OR berry OR blackber* OR blueber* OR "bok choy" OR brassica* OR broccoli OR cabbage* OR cantaloupe* OR carrot* OR cauliflower OR celer* OR chard OR chervil OR chicory OR cilantro OR collard* OR coriander OR cress OR cucumber* OR current* OR dill OR endive OR garlic OR gourd OR honeydew* OR kale OR kohlrabi* OR leek* OR lettuce* OR melon* OR mesclun OR mint OR mizuna OR mulber* OR mustard OR onion* OR oregano OR "pak choi" OR parsley OR pepper* OR radicchio OR radish OR raspber* OR rocket OR savory OR shallot* OR spinach OR sprout* OR squash* OR strawber* OR tomato* OR turmeric* OR turnip* OR watercress OR watermelon* OR zucchini*)
	AND
Hydroponics	TS=(aeroponic* OR aquaponic* OR "controlled environment" OR hydroponic* OR "nutrient film technique*" OR "nutrient solution*" OR soilless OR soil-free)

E. Food Science and Technology Abstracts (via EBSCOhost)

Block	Terms
Human pathogen	TS=(campylobacter OR clostridium OR coliform* OR cryptosporidium OR cyclospora OR e.coli OR "e. coli" OR enterobacter* OR escherichia OR giardia OR hepatitis OR listeria OR non-o157 OR norovirus* OR norwalk OR o157 OR salmonella OR shigella OR staphylococcus OR stec OR toxoplasma OR vibrio OR yersinia)
	AND
Fresh produce	TS=(vegetable* OR produce OR leafy OR green* OR microgreen* OR micro-green* OR babygreen* OR "spring mix" OR salad* OR herb* OR sprout* OR artichoke OR arugula OR basil OR berries OR berry OR blackber* OR blueber* OR "bok choy" OR brassica* OR broccoli OR cabbage* OR cantaloupe* OR carrot* OR cauliflower OR celer* OR chard OR chervil OR chicory OR cilantro OR collard* OR coriander OR cress OR cucumber* OR current* OR dill OR endive OR garlic OR gourd OR honeydew* OR kale OR kohlrabi* OR leek* OR lettuce* OR melon* OR mesclun OR mint OR mizuna OR mulber* OR

	mustard OR onion* OR oregano OR "pak choi" OR parsley OR pepper* OR radicchio OR radish OR raspber* OR rocket OR savory OR shallot* OR spinach OR sprout* OR squash* OR strawber* OR tomato* OR turmeric* OR turnip* OR watercress OR watermelon* OR zucchini*)
	AND
Hydroponics	TS=(aeroponic* OR aquaponic* OR "controlled environment" OR hydroponic* OR "nutrient film technique*" OR "nutrient solution*" OR soilless OR soil-free)

Table S2. Criteria and definitions for Relevance Screening (RS1 and RS2) in identifying and characterizing articles on hydroponic food safety

**Relevance screening of titles and abstracts (RS1)**		
**Question**	**Options**	**Definitions/additional notes**
RS1-Q1. Does the study (abstract) investigate any food safety issues in hydroponic fresh produce?		Exclusion criteria level:<br>1. Not food safety<br>2. Not fresh produce<br>3. Not hydroponic production
	Yes (include)	All studies that investigated human pathogen for microbial food safety. Human pathogens are any bacteria, viruses, or parasites that cause foodborne diseases.
	Not clear (include)	Fresh produce is any fruit and vegetable that has not been processed in any manner, commonly consumed raw.
	No (exclude)	Hydroponic produce production is the series of steps starting from seeding to harvesting where crops are grown in soilless environment usually in nutrient-rich solution.
RS1-Q2. Is this abstract primary research		Primary research represents a study where the authors collected and analyzed their own data – may use quantitative or qualitative methods or both to investigate the research question and report original results.

	Yes, primary research (include)	
	No, systematic review /meta-analyses (exclude)	Any systematic review or meta-analysis that identifies, appraises, and summarizes research findings.
	No, literature review or commentary (exclude)	A comprehensive or brief narrative review or commentary (from peer-reviewed articles to lay magazine or newspaper articles or briefs) describing and/or discussing the issue of microbial food safety.
	No, risk assessment, predictive model or economic assessment that does not include primary data (exclude)	
	No, other, specify: ________	

Relevance screening of full articles (RS2) - Only proceed to RS2 if you answered Yes to Q1 & 2 (RS1)

**Question**	**Options**	**Definitions/additional notes**
1. What is the study design? (check all that applies)		
	Prevalence and occurrence study	Prevalence & occurrence studies: studies into incidence, or presence or occurrence of microorganism at a particular period of time or the proportion (part/ amount) of hydroponic crop or environment contaminated with human pathogens within a (single point) time and location frame.
	Intervention study	Intervention (mitigations) studies addressing conditions and practices (behaviors or biologic factors), management practice, strategy) that prevent microbial food safety hazards for fresh produce (intervention /mitigation practices)

	Risk factor study	Risk factor studies: investigate pre- or post- harvest management practices, human behaviors in the production chain, or biologic factors related to the hydroponic fresh produce farming that may increase the chances/incidence of contamination at any of the fork to farm stage.
	Methodology study	Methodology studies: investigate rapid or other detection approaches to be implemented in hydroponic food safety.
	None of the above (exclude)	
2. Does the study describe any of the following?<br><br>(check all that applies)		
	Internalization study	Pathogen internalization studies -examine the ability of pathogen to penetrate the internal tissues of hydroponic crops.
	Survival study	Survival of human pathogen: investigate the survival, detection, persistence of microbial agents within a hydroponic crop system, including hydroponic environment at any stage of production.
	Microbial quality study	Cross sectional studies investigating bacterial load on fresh produce and presence or absence of human pathogens.
Only proceed if you answered Yes to RS2 Q1 otherwise submit form		
2. What pathogen was investigated?<br><br>(check all that apply)		Human pathogens are any bacteria, viruses, or parasites that cause foodborne diseases. Pathogens are microbes

	that harm their host and cause symptoms we recognize as an infectious disease.
**Bacteria**	
Campylobacter spp.	
Coliform/ Coliforms	
Clostridium spp.	
E. coli generic	
E. coli surrogate strain	
Listeria monocytogenes	
Listeria spp.	
Salmonella spp.	
Shigella spp.	
Staphylococcus aureus	
STEC non-O157	
STEC O157	
Vibrio parahaemolyticus	
Yersinia spp.	
**Parasite**	
Cryptosporidium spp.	
Cyclospora	
Giardia spp.	

	Toxoplasma gondii	
	**Virus**	
	Hepatitis A	
	Hepatitis E	
	Norovirus	
	Norwalk	
	Other, please specify___	
	No human health related organism investigated (exclude)	
3. What fresh fruit or vegetable type was investigated?<br>(check all that apply)		Fresh produce is any fruit and vegetable that is commonly consumed raw (FDA definition)
	**Leafy greens**	Exclude: non-produce items such as coffee, juice, guacamole, etc.
	Arugula	
	Basil	
	Cabbage	
	Chard	
	Celery	
	Chervil	
	Chicory	
	Cilantro	

	Coriander
	Dill
	Frizee
	Green Leaf lettuce(s)
	Iceberg
	Iceberg Lettuce
	Kale
	Leafy greens
	Leafy salad
	Lettuce
	Mesclun
	Mint
	Mustard Greens
	Parsley
	Radicchio
	Savory
	Salad greens
	Salad vegetable(s)
	Spinach
	Sprout

	"Spring Mix"	
	Watercress	
	**Melons**	
	Cantaloupe	
	Honeydew	
	Watermelon	
	Melons	
	**Root and seeded Vegetables**	
	Tomato	
	Pepper	
	Cucumber	
	Squash	
	Onion	
	Other, please specify___	
	No produce investigated (exclude)	
4. What hydroponic system was investigated in this research? (check all that apply)		Hydroponic systems are systems for growing crops in nutrient-rich solution. The system uses either organic or inorganic substrate to support plant growth
	NFT	Nutrient Film Technique
	DWC	Deep Water Culture
	Drip system (Dutch bucket/Gutters)	

	Ebb and Flow<br>Aquaponic<br>Aeroponic<br>“Hydroponic type not specified.<br>Other, please specify___<br>Non specified<br>Unknown	
5. What stem in produce value chain is the study relevant to?<br>(check all that apply)	Preharvest<br>Harvest<br>Post harvest handling<br>Storage<br>Retail<br>Foodservice (restaurant, institutional, catering)<br>Consumer handling (household preparation)<br>Other, please specify___	Step in the farm to fork hydroponic value chain includes all the stages that hydroponic produce goes through, from growing the plants to being eaten in homes.
6. What food safety risk area(s) were investigated in this study?		Thematic/operational risk area are the activities, factors and practices that is most likely contributing to microbial contamination.

(heck all that apply)		
	Water management<br>Worker hygiene<br>Environmental and equipment sanitation<br>Waste management<br>Post harvest handling<br>Animal intrusion<br>Traceability<br>Other, please specify___	
What intervention(s) were investigated in this study?<br>check all that apply)		Any chemical, biological, physical, or behavioral management practices to control or eliminate human pathogens.
	Sodium hypochlorite<br>Chlorine dioxide<br>Ozone<br>Hydrogen Peroxide<br>Irradiation<br>Peracetic Acid<br>UV<br>Aqueous chlorine<br>Quaternary Ammonium	

Other, please specify___

Table S3. Data extraction and Quality Assessment tool to map intervention studies related to hydroponic production.

**Second level relevant screening (RS2)**		
**Questions**	**Options**	**Definitions/additional notes**
RS2 - Q1. What is the Covidence_ID#		ID# Please use Covidence ID generated from the software
RS2 - Q2. Data Extraction user		Text_Name of the person who is extracting the data
RS2 - Q3. Verification user		Text_Name of the person who is verifying the data
RS2 - Q4. Does this study investigate food safety intervention in hydroponic produce?	Yes	Study contains relevant data on intervention or mitigation practices/strategies that will prevent, eliminate, or minimize contamination of fresh produce
	No	Study does not contain relevant data related to intervention or mitigation practices/strategies of fresh produce.
**Only proceed to with the extraction if you answered Yes to RS2 - Q4.**		
**Questions**	**Options**	**Definitions/additional notes**
1. Did the article declare any conflict of interest	Yes	Conflicts of interest in research setting is the situation where an individual or organization has financial, personal, or professional ties to research that could compromise their objectivity or integrity of the research outcome.
	No	COI disclosure form
2. What pathogen was investigated?		Human pathogens are any bacteria, viruses, or parasites that cause foodborne diseases. Pathogens are microbes that harm

		their host and cause symptoms we recognize as an infectious disease.
(check all that apply)	**Bacteria**<br>Campylobacter spp.<br>Coliform/ Coliforms<br>Clostridium spp.<br>E. coli generic<br>E. coli surrogate strain<br>Listeria monocytogenes<br>Listeria spp.<br>Salmonella spp.<br>Shigella spp.<br>Staphylococcus aureus<br>STEC non-O157<br>STEC O157<br>Vibrio parahaemolyticus<br>Yersinia spp.<br>**Parasite**<br>Cryptosporidium spp.<br>Cyclospora<br>Giardia spp.<br>Toxoplasma gondii<br>**Virus**<br>Hepatitis A<br>Hepatitis E<br>Norovirus<br>Norwalk<br>Other, please specify___<br>No human health related organism investigated (exclude)	

3. What fresh fruit or vegetable type was investigated?<br>(check all that apply)		Fresh produce is any fruit and vegetable that is commonly consumed raw (FDA definition)
	**Leafy greens**	Exclude: non-produce items such as coffee, juice, guacamole, etc.
	Arugula	
	Basil	
	Cabbage	
	Chard	
	Celery	
	Chervil	
	Chicory	
	Cilantro	
	Coriander	
	Dill	
	Frizee	
	Green Leaf lettuce(s)	
	Iceberg	
	Iceberg Lettuce	
	Kale	
	Leafy greens	
	Leafy salad	
	Lettuce	
	Mesclun	
	Mint	
	Mustard Greens	
	Parsley	
	Radicchio	
	Savory	
	Salad greens	
	Salad vegetable(s)	

	Spinach
	Sprout
	“Spring Mix”
	Watercress
	**Melons**
	Cantaloupe
	Honeydew
	Watermelon
	Melons
	**Root and seeded Vegetables**
	Tomato
	Pepper
	Cucumber
	Squash
	Onion
	Other, please specify___
	No produce investigated (exclude)
4. Did this study investigate chemical intervention?	Yes
	No
4a. What kind of chemical intervention or mitigation strategies were implemented in the study to address food safety concerns in hydroponic production? (check all that apply)	Chemical
	Sodium hypochlorite
	Peroxyacetic acid
	Chlorine dioxide
	Hydrogen Peroxide
	Aqueous Chlorine
	Quaternary ammonium

	Acetic acid
	Erythorbyl laurate
	pH
	Ozone
	Disinfectant
	Essential oils
	Other, please specify___
5. Did this study investigate physical intervention?	Yes
	No
5a. What kind of physical intervention or mitigation strategies were implemented in the study to address food safety concerns in hydroponic production?	UV treatment
(check all that apply)	Irradiation
	Filtration
	High hydrostatic pressure (HHP)
	Photodynamic inactivation (PDI)
	Heat treatment
	Hot air treatment
	Electrolyzed water
	Electro Beam Irradiation
	Other, please specify___
6. Did this study investigate biological intervention?	Yes
	No
6a. What kind of biological intervention or mitigation strategies were implemented in the study to address food	Protozoa

safety concerns in hydroponic production?<br>(check all that apply)	Protective culture<br>Other, please specify___	
7. Did this study investigate behavioral intervention?	Yes<br>No	
7a. What kind of behavioral intervention or mitigation practices were implemented in the study to address food safety concerns in hydroponic production?<br>(check all that apply)	Hand washing<br>Personal Protective Equipment (PPE)<br>Good Agricultural Practices trainings<br>Produce Safety Alliance (PSA) training<br>Other, please specify___	
8. At which point in the value chain was the interventions implemented?<br>(check all that apply)	Propagation (Nutrient preparation, sowing, raising seedlings)<br>Transplanting<br>Pre harvest production<br>Harvest<br>Post harvest handling<br>Transportation<br>Storage<br>Retail<br>Other, please specify___	
9. What hydroponic system was used in this study?<br>(check all that apply)	Not reported<br>Aquaponic	Not reported: the study was done under hydroponic system but is not reported the type of hydroponic system.<br>Vertical farming: This involves growing crops in vertical multi-level racks inside a controlled-environment building. Each rack has space above that allows vegetation to grow

	upwards. These high-tech farms drastically reduce the space needed to grow foods, as they can be housed in warehouses, industrial buildings or even skyscrapers rather than using farmland or traditional greenhouses.
Nutrient Film Technique	Indoor farming: Is a type of soilless culture where crops are grown in a single level or floor and may use natural or artificial lighting.
Deep Water Culture (DWC)	Aquaponics: This is a type of CEA that combines hydroponics with aquaculture, where fish and plants are grown together in a closed system.
Dutch bucket	Aeroponics: This involves growing crops in an air-based environment, with roots suspended in a mist of nutrient-rich water. Aeroponics systems suspend plants in the air and expose the naked roots to a nutrient-filled mist. Aeroponics systems are enclosed frameworks, like cubes or towers, that can hold a multitude of plants at once. Water and nutrients are stored in a reservoir, and then pumped to a nozzle that atomizes the solution and distributes it as a fine mist. The mist is usually released from the top of the tower, allowing it to cascade down the chamber. Some aeroponics continuously mist the plant's roots, much like NFT systems always expose the roots to the nutrient film. Others function more like the ebb and flow system, spraying the roots with mist in intervals. Aeroponics do not need substrate media to survive. The root's constant exposure to air allows them to drink in oxygen and grow at an accelerated rate.
Gutters	Nutrient Film Technique (NFT): suspend plants above a stream of continuously flowing nutrient solution that washes over the ends of the plant's root systems. The channels holding the plants are tilted, allowing water to run down the length of the grow tray before draining into the reservoir below. The water in the reservoir is then aerated via air stone. A submersible pump then pumps the nutrient-rich water out

	of the reservoir and back to the top of the channel. The nutrient film technique is a recirculating hydroponic system.
Aeroponic	Deep Water Culture (DWC): also known as DWC systems. are one of the easiest and most popular methods of hydroponics on the market. This system consists of a deep reservoir filled with nutrient solution with an air pump to keep the water aerated. The plants are suspended on a growing bed, leaving the roots submerged in the solution, providing it with perpetual access to nutrition, water, and oxygen.
Ebb and flow (flood and drain)	Drip System: the aerated and nutrient-rich reservoir pumps solution through a network of tubes to individual plants. This solution is dripped slowly into the growing media surrounding the root system, keeping the plants moist and well-nourished. Drip systems are the most popular and widespread method of hydroponics, especially among commercial growers. Drip systems can be individual plants or massive irrigation operations. There are two configurations of drip system hydroponics: recovery and non-recovery. In recovery systems, more popular with smaller, at-home growers, the excess water is drained from the grow bed back into the reservoir to be recirculated during the next drip cycle. In non-recovery systems, the excess water drains out of the growing media and runs to waste.
Wick system	Ebb & Flow: work by flooding a grow bed with a nutrient solution from a reservoir below. The submersible pump in the reservoir is equipped with a timer. When the timer starts, the pump fills the grow bed with the water and nutrients. When the timer stops, gravity slowly drains the water out of the grow bed and flushes it back into the reservoir. While the grow bed is flooded, the plants drink up the nutrient solution through their root systems. When the water ebbs and the grow bed empty, the roots dry out. The dry roots then oxygenate in the interval before the next flood. The length of

		time between floods is dictated by the size of your grow bed and the size of your plants.
	Drip system	Wick System: plants are nestled in growing media on a tray that sits on top of a reservoir. This reservoir houses a water solution with dissolved nutrients. Wicks travel from the reservoir to the growing tray. Water and nutrients flow up the wick and saturate the growing media around the root systems of the plants. These wicks can be made of material as simple as rope, string, or felt. Wick systems are by far the simplest form of hydroponics. Wick systems are passive hydroponics - meaning they don't require mechanical parts like pumps to function.
	Other, please specify___	
10. What type of CEA structure was used in this study? (check all that apply)	Not reported	
	Indoor Farming	
	Glass/polycarbonate greenhouse	
	High Tunnels	
	Screen House	
	Shade House	
	Retail growth chamber	
	Other, please specify___	
11. What type of facilities were used for the hydroponic CEA research study? (check all that apply)	Not reported	
	Commercial postharvest facility	
	Commercial greenhouse/facility	
	Indoor facilities	
	Research greenhouse/ facility	
	Research indoor growing facility	
	Research Aquaponic facility	
	Other, please specify___	

12. What type of sample was collected in the study to assess the intervention's effectiveness?	Not reported	Edible leaf/fruit: The fresh mass of produce that is used to be sell in the market for consumption and was weighed and analyzed for microbial count and intervention effect.
(check all that apply)	Edible leaves/fruit	**Edible leaf/fruit:** The fresh mass of produce that is used to be sell in the market for consumption and was weighed and analyzed for microbial count and intervention effect.
	Non-edibe leaves/flowers	Non-edible leaves/flowers: the fresh mass of produce that doesn't go to the market and is not sell for consumption but is weighed and analyzed for microbial count and intervention effect.
	Root	Root: The root of produce that was weighed and analyzed for microbial count and intervention effect.
	Substrate (please specify)	Substrate: The type of substrate for production of produce analyzed for microbial count and intervention effect. Please specify_ type the substrate that was used in the system (e.g., rockwool, perlite, peatmoss, vermiculite, coconut oil, smart gravel, others)
	Nutrient solution	Nutrient solution: The water culture that contains the nutrient content that was collected and analyzed for microbial count.
	Hydroponic surfaces	Hydroponic surfaces: The surfaces that are part of the hydroponic system and have contact with the edible and non-edible parts of the produce.
	Facilities surfaces	Facilities surfaces: The surfaces that are not part of the hydroponic system but are close to where the produce is managed (the indirect surfaces that can be around the produce).
	Whole plant	
	Other, please specify___	
13. What was the frequency of application for intervention/mitigation practices?	Not reported	
(check all that apply)	One time application	One time application: the intervention treatment was applied one time during the research study period.

	Multiple applications overtime	Multiple applications overtime: the intervention treatment was applied every certain period (e.g., hourly, daily, weekly, other).
	Multiple interventions implemented at the same time	Multiple interventions implemented at the same time: multiple interventions treatments were used one time during the research study period.
	Multiple interventions implemented successively	Multiple interventions implemented successively:
	In-line intervention (consistent injection or exposure)	In-line intervention (consistent or exposure):
	Other, please specify___	
14. How was the effectiveness of the intervention or mitigation practices measured?	Not reported	
	Concentration one time after treatment	Concentration one time after treatment: the sample to measure the intervention treatment effectiveness was taken one time after treatment during the research study period.
	Concentration overtime after treatment	Concentration overtime after treatment: concentration measured multiple times every X time.
	Prevalence one time after treatment	Prevalence one time after treatment: prevalence measured one time.
	Prevalence overtime after treatment	Prevalence overtime after treatment: prevalence measured multiple times every X time.
	Behavior before and after treatment	Behavior before and after treatment:
	Other, please specify___	
15. Is raw or unadjusted data provided?	Yes	Yes: the following data must be reported. Numerator and denominator, or: Proportion + EITHER numerator or denominator . For measures of association/effect:OR/RR/IR/RD reported and its measure of variability (SE, SD, CI) or P-value is provided. For continuous measures: Mean value, sample size, and SD, Mean value and SE/CIs
	No	Graphical data only, No reporting of raw results, Just median, Only p-value, Only numerator, Only denominator

16. What kind of control was used for the research study?<br>(check all that apply)	Not reported<br><br>Non-inoculated control<br>Inoculated control<br>Non-treated control<br>Treated control<br>Other, please specify___	
17. How many times was the experiment replicated?	Not reported<br><br>Single replicate<br>Duplicate<br>Triplicate<br>Other, please specify___	The experiment was replicated as: single replicated, duplicate or triplicate.
18. Was the sample processed in replicates?	Not reported<br><br>Single replicate<br>Duplicate<br>Triplicate<br>Other, please specify___	The sampling was replicated as: single replicated, duplicate or triplicate
19. Were the methods described adequately to be reproduced?	Yes, adequate description to be replicated<br>Insufficient	The methods were reported in sufficient detail to understand the methodology to be replicated<br>Methods and adjustments are not clear, or some details are missing.
20. What was the effectiveness of the intervention as reported by the authors?	Log reduction<br><br>Concentrations<br>Prevalence<br><br>Not reported<br>Other, please specify___	Log reduction: text specify log reduction.<br><br>Concentrations: text specify concentration after treatment<br>Prevalence: text how many __ of group of X are contaminated at X time.
21. What is the Covidence_ID#		ID# Please use Covidence ID generated from the software

22. Comments	Text	Provide any additional comments if necessary
